# Round the Hospitals

**Published:** 1919-08-16

**Authors:** 


### ROUND THE HOSPITALS.

On August 7 a Supplement to the *Gazette* contained the names of a large number of ladies to whom the King has been pleased to award the Royal Red Cross in recognition of valuable nursing services under the British Red Cross Society and the Order of St. John of Jerusalem in England. Bar to the Royal Red Cross, Miss L. Cushion, R.R.C., matron, B.R.C.S. Hospital, Netley; Miss M. C. Fisher, matron, B.R.C.S.; Miss A. M. McDonnell, R.R.C., matron, Red Cross Auxiliary Hospital, Perth. Royal Red Cross, First Class: Miss L. Boot; Miss E. E. Jones, A.R.R.C.; Miss M. F. Lightfoot, A.R.R.C.; Miss K. S. MacGregor; Mrs. M. C. B. Millar, A.R.R.C.; Miss L. Ruscoe, A.R.R.C.; Miss R. M. Skipworth, A.R.R.C.; Miss E. Stevenson, A.R.R.C.; Miss C. M. K. Tennyson-Smith, A.R.R.C.; Mrs. L. Wilson, A.R.R.C.; Miss J. Wyse. All the above were matrons or sisters in charge of Red Cross Auxiliary Hospitals. The King has awarded the Second Class of the R.R.C. to between 300 and 400 ladies, matrons, sisters, staff nurses, commandants, and V.A.D. nurses in Auxiliary Hospitals. The King has also awarded the R.R.C., Second Class, to Miss H. Burton, Miss R. S. Cumming, Miss R. Gilmer, Miss E. Grant, and Miss E. M. McCullum, matrons in charge of hospitals in New Zealand, and to Miss A. Davis, matron, Adana Hospital.

---

The commemoration of Dame Maud McCarthy's great work in France as Matron-in-Chief during the whole war is one into which all Q.A.I.M.N.S. nurses who have served on the Western Front must have thrown themselves whole-heartedly. She not only won their love in a remarkable degree, but by her own ardour inspired them at all times and under all circumstances to "carry on." It is not surprising that their appreciation of these qualities and of her untiring and unselfish devotion should lead them to make some permanent commemoration of their personal feelings towards their chief. This has taken the form of the purchase of an annuity for the benefit of a nurse in the Trained Nurses' Annuity Fund, to be called the "Dame Maud McCarthy Annuity," to which Dame Maud has the right to nominate an annuitant. She has from the very beginning of the War realised the need for annuity funds on behalf of disabled trained

**ROUND THE HOSPITALS**—(*continued*).

nurses, and there were few if any gifts which she would appreciate more fully. In this view the members of the Service have been confirmed by Dame Maud herself, who in thanking the Acting Principal Matrons of the bases and Army areas as representatives of the Nursing Service in France and requesting them to thank in her name all members of the Service, states she is deeply touched that they should have desired to connect her name with an object which she has always had so much at heart.

An illuminated address on parchment, bound in soft red leather, bearing in gold letters the name of Dame Maud McCarthy, G.B.E., R.R.C., has been presented to her recording this commemoration. It is signed by M. L. Rannie, M. M. Bond, M. M. Tunley, A. L. Walker, L. E. Mackay, G. M. Allen, E. C. Fox, A. Rowe, and E. J. Minns, all of Q.A.I.M.N.S. It records the appreciation of the members of the Nursing Services who have served under Dame Maud in France 1914 and 1919, their desire to show in some way the measure of that appreciation, and states that to commemorate her great work a voluntary subscription was raised which has been used for the purpose above set out. It concludes by expressing the pleasure it would give to all Dame Maud's B.E.F. staff if she would consent to the arrangement and wishes her every happiness and success in the future. It is interesting to note that as early as 1915 subscriptions from France were sent to the Trained Nurses' Annuity Fund, and further subscriptions have been sent regularly.

For centuries past it has been the custom of both Houses of Parliament, so Lord Curzon reminded the Lords, in the concluding stages of a great national struggle to offer a vote of congratulation and thanks to those brave men who upheld the honour, maintained the welfare, and added to the glory of the nation. The occasion, on August 6, when this tribute was voted in the two Houses was rendered memorable to the women of this country by their inclusion in the ranks of the patriots to whom the nation's thanks are due. The Prime Minister moved, amidst general applause: "That the thanks of this House be accorded to the women of the medical and other auxiliary services for their devotion in tending the sick and wounded, and for other duties faithfully and bravely discharged." Subsequently he spoke with feeling of the women who in great peril nursed the wounded and saved thousands of lives by their tender care, and declared that their achievements, with those of the other women of the auxiliary services, were written deep on the hearts of the people of this country. We believe with Lord Curzon that this form of tribute, which represents the organised sentiments of the nation, is more valued by its recipients than the shouts of the populace or even honours from the Crown.

The Air Ministry announces that members of women's formations who have been employed under a direct contract of service with the Royal Air Force Medical Service should apply to the Secretary (D.M.S.) Air Ministry for their two inches of the British War Medal riband. It is to be understood that receipt of the riband does not confirm the title of the recipient to the subsequent award of the medal.

Mr. Churchill announced, in answer to a question by Mr. Rawlinson in the House of Commons, that two hospital ships are being used to evacuate patients from the military hospitals in North Russia. There is an adequate supply of doctors and hospital requisites at both Archangel and Murmansk. Six British nurses are doing duty at Murmansk. There are no British nurses stationed at Archangel.

The Metropolitan Asylums Board is one of the largest employers of nurses in the kingdom, and the executive have shown, not for the first time, their power of seeing nursing questions from the point of view of nurses themselves. Considerable difference of opinion exists among matrons and nursing sisters in regard to the best system of shortened hours to adopt, and in the circumstances nothing could be wiser than to refer the matter to the persons who will be principally concerned. Accordingly it has been arranged that a direct ballot of the nursing staff is to be taken for choice between two schemes, one based on a forty-eight hours' and the other on a fifty hours' working week. Other points to be decided by ballot are whether or not the nursing staff shall be paid at inclusive cash rates, subject to deductions for emoluments received in kind, and whether the payments shall be weekly or monthly. The result of the ballot will be awaited with considerable interest.

A telegram has been received by the Under-Secretary of State for Foreign Affairs, reporting a complete breakdown of medical and nursing arrangements in the Budapest hospitals. Dr. Hilda Clark, Dr. Hector Munro, and Mr. Buxton have arrived there, and report that the position is desperate. Only paper dressings are available. There are no general anæsthetics, and a great scarcity of all medicines, soap, and linen.

There continues to be much distress among demobilised sisters. The Nurses' Demobilisation Employment Bureau at 16 Curzon Street has dealt with 3,000 cases, of which less than half have been placed, while over 1,000 on the books are still seeking posts. The Council of the College of Nursing are taking active steps to deal with the situation, and have written to the Ministry of Pensions, calling attention, not only to the number of unemployed nurses, but also to the numerous cases where the nursing sisters have been incapacitated by their exertions from following their profession again. The Council are of opinion that a scheme should be formulated by the Ministry of Pensions which shall deal adequately with the question, and point out that gratuities merely tide over a short time, and leave

**ROUND THE HOSPITALS—*(continued)*.**

the real problem of the nurse's future unsolved. It is a mistake to suppose that Army sisters will not accept any except positions of authority. So far as their strength will permit, many are willing to accept almost any post.

---

THE agitation against the fourth year of training continues in Melbourne. Either the probationers who agitate against it are strangely ignorant and misguided, or the hospital managers have failed to demonstrate satisfactorily its value in the scheme of training. It has to be clearly recognised that not every hospital is able to provide a fourth year's course of sufficient value to compensate the probationer for the postponement of her full-earning period. A deputation of nurses in training at metropolitan hospitals (Melbourne) waited on the Premier (Mr. Lawson) on May 22 and stated their views on several points. They asked for the abolition of the fourth year, as, according to examinations, nurses can be fully trained in two and a-half years. Those who wish to qualify in special subjects, after four years in a general hospital, have to spend as long a time in training as does a doctor. The fourth year also postponed their earning power. As qualified nurses outside they could earn £3 3s. a week, while in hospital they received only £40 per annum. The deputation also asked for an eight-hour day, as the wardmaids, the dispensers, and the clerks had, and pointed out that excessive fatigue impairs efficiency. The Premier was sympathetic, and it is likely that during the next session of the State Parliament some reforms on these lines will be effected.

---

GERMAN nurses have not always distinguished themselves during the war by humane treatment of their enemies, and it is all the more desirable to bring to light occasions on which they have acted up to the professional standard. In the distressing evidence given during the court-martial on Assistant Surgeon Fratel, I.M.D., for brutal treatment of the British sick prisoners in the Bagtsche Hospital, it transpired that two German nurses had openly protested. The sick and dying men were left for hours lying on the ground outside the hospital. The British patients were in a filthy and neglected condition, so much so that these German nurses complained that it was the most disgusting thing they had ever seen, and that if the British were not shifted from these terrible quarters they would leave Bagtsche and return to Constantinople. One witness, in charge of a cholera tent, stated that the only medicine he could secure for the men was some quinine he got from a German nurse.

---

THE Weekly Board of the Middlesex Hospital announce in the annual report that a scheme is under immediate consideration by which the nursing staff will benefit largely both as regards remuneration and hours of work. The closing of the hospital for three months from July 1 will enable this scheme to be worked out with full deliberation, and we anticipate that the new order of things will commence at the re-opening on October 1. Many generous donations have been received towards the new nurses' home and the alterations in the old ones, but a large sum is still required, and the Chairman pleads for a prompt response to the appeal for this object set on foot by H.R.H. Princess Alice, Countess of Athlone.

---

A PLEASANT ceremony took place at the meeting of Central Midwives Board on July 24, when an address was presented to Mr. Duncan, the retiring Secretary, from present and former members of the Board, together with a handsome chest and mirror. The Chairman reminded his hearers that Mr. Duncan had been Secretary before the Act came into force, and took ante-natal interest in its embryonic existence. He paid a high tribute to his unfailing courtesy and patience and prompt readiness to help. The new Secretary is Mr. Herbert George Westley, M.D.

---

MR. STEPHEN PAGET, who has examined the nurses at Edmonton Infirmary for some years in regard to their knowledge of nursing, has informed the Guardians that he is retiring from this work. In a letter to the Medical Superintendent (Colonel Mort), Mr. Paget states that it has always been a pleasure to examine nurses so admirably well taught and trained. The Guardians have decided to ask Professor Carless, of King's College and University of London, to conduct the examinations in future. A gold medal is to be awarded to the "top" nurse of each year for proficiency.

---

AT the first annual meeting of the newly formed Durham County Nursing Association, which took place at the Shire Hall, Durham, notice was given that by arrangement with the Durham County Council a grant of £30 annually, and further help if necessary, would be granted to District Associations employing an approved certified nurse-midwife. The County Council will also make grants for the nursing of tuberculosis and certain other cases. These grants will greatly facilitate the work of the County Association. In many parts of Durham the population is dense, and housing conditions are very bad. It is not surprising to find that infant mortality is higher than in other counties, and the problems which confront the young association are not easy ones. But already generous financial help is forthcoming, and the future outlook is promising.

---

AT the inquest, held at Liverpool on August 6, on the body of Nurse Alice Kate Jones, of the David Lewis Northern Hospital, a verdict of wilful murder was returned against Joseph Boy Hutty, formerly a Canadian soldier, who was treated at the hospital last year for shell-shock. Mr. Robert R. Jones, father of the nurse, stated a friendship sprang up between his daughter and Hutty, who with his approval spent a week at his house last

**ROUND THE HOSPITALS**—*(continued)*.

November. He was in a weak state, and could not walk without help, while he had no friends in England. Before he left for America he spoke to witness about being engaged to Miss Jones, and was told he had better wait till he was established in business. Later on, in February, Nurse Jones wrote to break off the engagement. On July 15 he wrote to her sister to say he had come over to try to make it up. Nurse Jones wrote herself, refusing to meet him, and on the night of July 24 was shot dead outside the hospital. She had previously had a letter from Hutty to inform her he was returning to America.

The Berkshire County Nursing Association has made such good progress during the fourteen years it has been in existence that the Chairman of the Executive Committee, Mrs. Mount, is able to report that only nine districts in the county lack a trained nurse. There are fifty-three District Associations affiliated to the County Association, and an effort is being made to raise sufficient funds to establish a central nurses' home in Reading, whence emergency nurses can be sent out to fill vacancies as they occur, and thus ensure continuity. There is no lack of good candidates for training, and we admire the courage which has prompted the Committee to train all the suitable women they can find, and borrow from the bank to provide the money. The spirit which directs the Association is admirable, and must quickly command that enthusiasm which turns to money. We think the County Council is not doing all it might to forward good nursing and strengthen the hands of the Association. The grant of £5 per 1,000 of the population available for maternity and child welfare work strikes us as pitiably inadequate under present conditions, though it was welcomed in pre-war days.

The Carnarvon Guardians have decided to ask the Local Government Board to hold an informal inquiry into the disagreements alleged to have subsisted for some years between the matron of the workhouse and the head nurse of the Eryri Hospital, which is under the Guardians' control. With the launching of the Health Ministry it may be permitted to hope that the state of affairs which has made such friction in many instances almost unavoidable, owing to the overlapping of the head nurse's powers in certain respects with those of the workhouse matron, will now definitely come to an end. This is no mere question of a personal dispute. It has its root far deeper, and the system needs radical alteration.

The eighty-third annual Court of Governors of the East Suffolk and Ipswich Hospital was an unusually notable event from a nursing point of view, for it marks the inauguration of a one day's rest in seven for the nursing staff and the termination of their exile to temporary quarters while the wounded were in occupation. The report alludes warmly to the devotion shown by all the nurses during the war, and lays down six principal duties which the authorities owe to the staff. First, to afford the best possible training, practical and theoretical, and test it by outside examiners; secondly, to afford them comfortable quarters; thirdly, to provide good and varied food; fourthly, to pay adequate salaries; fifthly, to arrange for regular annual holidays and frequent off-duty times; sixthly, to provide for healthy recreation and general comforts. In all respects except that of quarters the Ipswich Hospital has carried out its programme, and the London examiner's report testifies to the high state of efficiency maintained under the matron, Miss Deane. It was announced that, owing to the generosity of Mr. Cobbold, who presented £1,000 for this purpose, an "East Suffolk and Ipswich Hospital Fund" has been founded for the benefit of nurses in need of help.

At the Glasgow Royal Infirmary fifty-eight nurses left on the completion of their training last year, the majority to take up some branch of war work. The honours accorded to Glasgow Infirmary-trained nurses were as follows: One Royal Red Cross, First Class; ten Royal Red Cross, Second Class; seven mentioned in despatches; one Croix de Guerre. The new badge struck in connection with the Royal Infirmary School of Nursing has been provided for and presented to the nurses completing their training, through the kindness of Mr. James S. Craig, one of the managers. It is very popular with former nurses, many of whom have applied for it, and will for the future be presented to each probationer on receiving her certificate. We learn with pleasure that the matron, Miss Melrose, has completely recovered from the severe strain which the exceptional fatigues of the last few years had entailed upon her.

A prolonged discussion took place at the last monthly meeting of the Burnley Board of Guardians over the recommendation of the House Committee to raise the salary of Miss Nugent, superintendent nurse at the infirmary, from £100 to £130 per annum, making with the war bonus of £20 a total remuneration of £150. Miss Nugent's high abilities, and in particular her devoted service during the war, elicited universal commendation, but the proposal met with opposition on the ground that if the recommendation were carried out her salary would be in excess of that given to the matron of the workhouse, who would thereby be placed in an inferior position. It was curious to notice that several of the Guardians clung to the delusion that the superintendent nurse was under the control of the matron, as well as of the master, of the workhouse. The recommendation to raise Miss Nugent's salary was at length passed. We note that a resident medical officer has been appointed at the Burnley Workhouse, and would suggest that the best thing to do would be to sever the infirmary altogether from workhouse control, and make it a training-school for nurses.

